# Supplementary material for: The interactome of intact mitochondria by cross-linking mass spectrometry provides evidence for coexisting respiratory supercomplexes
Source: Mol Cell Proteomics. 2017 Dec 8;17(2):216–32. doi: 10.1074/mcp.RA117.000470 (PMC5795388; doi:10.1074/mcp.RA117.000470)
Supplement: Supplemental Data [file supp_RA117.000470_133922_0_supp_23978_fzffwf.zip › spectra_annotation/mito_DR_spectra_annotation/345-1-1-1-14-1.pdf]

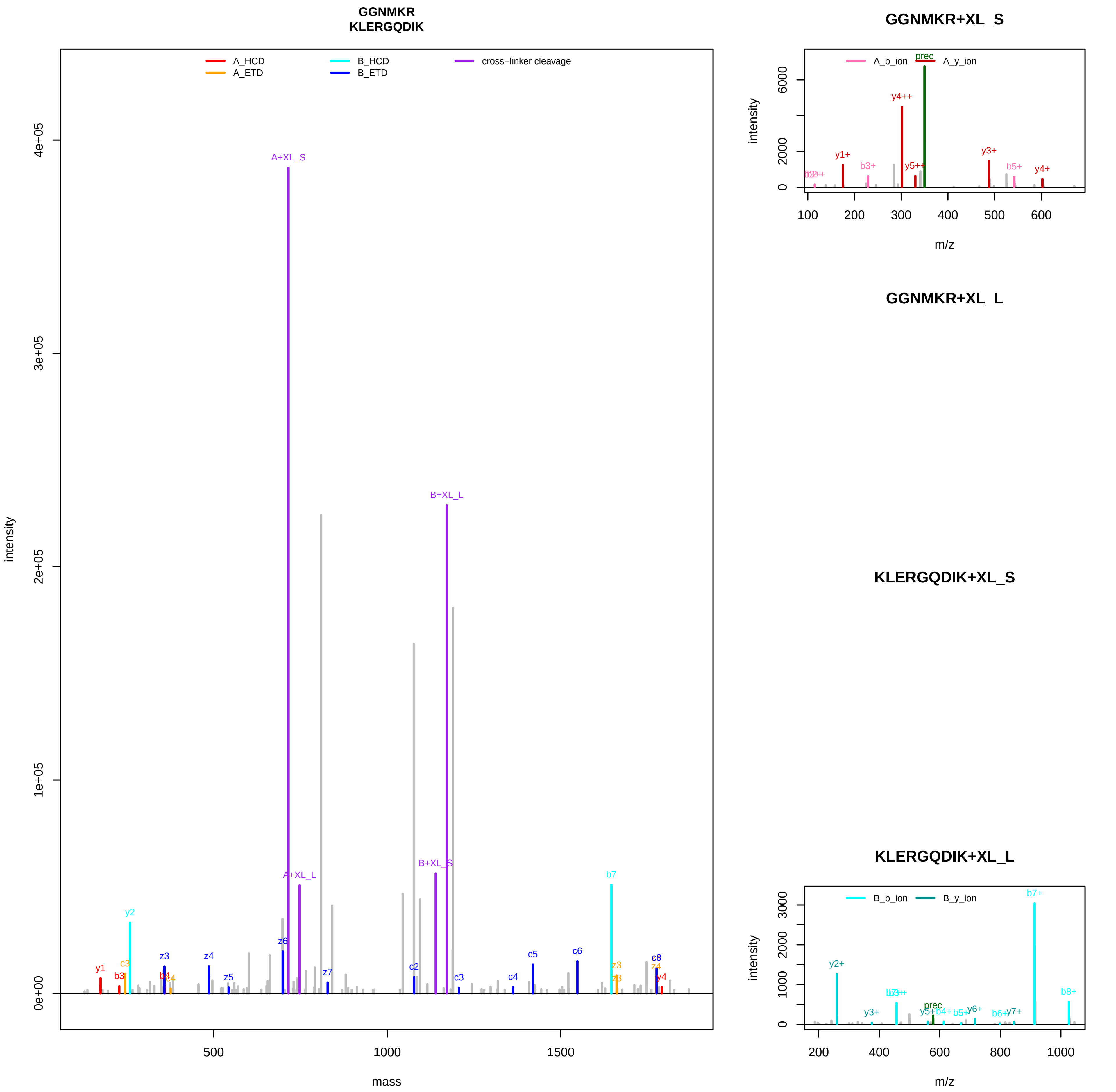
GGNMKR
KLERGQDIK
A_HCD
A_ETD
B_HCD
B_ETD
cross−linker cleavage
A+XL_S
B+XL_L
A+XL_L
B+XL_S
intensity
mass
0e+00
1e+05
2e+05
3e+05
4e+05
500
1000
1500
y1 b3 c3 y2 z3 b4 z4 z5 z6 z7 c2 c3 c4 c5 c6 b7 z3 c8 z4 y4
GGNMKR+XL_S
A_b_ion
A_y_ion
prec
y1+ b3+ y4++ y5++ y3+ b5+ y4+
m/z
GGNMKR+XL_L
KLERGQDIK+XL_S
KLERGQDIK+XL_L
B_b_ion
B_y_ion
y2+ y3+ b7+ b8+ y5+ b4+ b5+ y6+ b6+ y7+
0
1000
2000
3000
200
400
600
800
1000
